# Correlation Analysis of Lower Limb Venous Dilation in Patients With Diabetic Foot Ulcers

**DOI:** 10.1155/jdr/2164138

**Published:** 2025-10-06

**Authors:** Rumei Li, Zhaoxi Li, Cuiman Chen, Xiaotong Zhu, Baoming Luo, Jing Tian

**Affiliations:** ^1^Department of Ultrasound, Sun Yat-sen Memorial Hospital, Sun Yat-sen University, Guangzhou, China; ^2^Central Laboratory, Dongguan People's Hospital/Affiliated Dongguan Hospital, Southern Medical University, Dongguan, China

**Keywords:** diabetic foot ulcer, lower limb venous dilation, procalcitonin, systolic blood pressure

## Abstract

**Background:**

Diabetic foot ulcers (DFUs) significantly contribute to disability and increased mortality rates among patients with diabetes. Researches on venous conditions of DFU are rather limited compared to other pathological factors. This study is aimed at investigating the correlation between venous dilation and various clinical and biochemical factors in patients with DFU.

**Methods:**

We enrolled 100 patients with DFU and performed Doppler ultrasound examinations of the lower extremity vascular system by a senior ultrasonographer. Clinical and biochemical characteristics were collected, and their correlation with venous dilation was analyzed by Spearman's correlation and multiple linear regression.

**Results:**

The diameters of the common femoral vein, femoral vein, great saphenous vein above knee and below knee (GSVa and GSVb), and the small saphenous vein (SSV) were larger on the affected side than those of the unaffected side of the ulcer, especially the superficial veins. Thus, the ratio of diameters of the superficial veins between affected and unaffected sides was calculated for correlation analysis. Fasting blood glucose levels were positively correlated to the ratio of GSVb, while plasma levels of procalcitonin and white blood cells, markers of inflammation, were found to be positively correlated with the ratio of SSV. However, after adjustment, male gender and duration of diabetes were the positive predictors for the changes in the ratio of GSVb, while systolic blood pressure (SBP) was an independent positive predictor for the changes in the ratio of SSV after adjustment. We did not identify a significant correlation between the severity of the ipsilateral dorsal pedis artery stenosis and the ratios of the GSV and SSV.

**Conclusion:**

Our findings indicate that venous dilation in the affected lower limb is a common occurrence in patients with DFU. Male gender, duration of diabetes, and SBP, but not localized arterial stenosis or serum inflammatory markers, were independent predictors for venous dilation below the knee. Understanding these correlations could contribute to the broader understanding of diabetes-related venous complications.

## 1. Introduction

Diabetic foot is a common chronic complication among patients with diabetes, and it is also one of the leading causes of disability and death [1]. It is reported that over 30% of patients with diabetes develop a foot ulcer during their lifetime, while about 20% of people with a diabetic foot ulcer (DFU) will undergo a lower extremity amputation [2]. In patients with diabetic foot, foot ulcers and deep tissue destruction, with or without infection, result from nerve abnormalities at the distal end of the lower limb and varying degrees of peripheral artery lesions [2, 3]. The venous condition of the lower limb in diabetic foot patients is a critical yet often overlooked aspect of diabetes management. Venous insufficiency, characterized by impaired venous return from the periphery, can exacerbate conditions like edema and ulceration, impairing wound healing by limiting oxygen and nutrient delivery [4].

Chronic venous diseases (CVDs), characterized by the impaired venous walls and valves, manifest as varicose veins, thrombosis, and even venous leg ulcers [5]. CVDs and diabetes are mainly seen as separate entities, but they often coexist. Clinical studies have revealed that diabetes was present twice in patients with CVDs as much as that observed in the general population [6]. In addition, early CVD was more frequently observed in patients with diabetes compared to the general population, although CVD severity did not differ significantly between the two groups [6, 7]. Whether venous insufficiency occurs before or after the occurrence of DFU is difficult to distinguish in most patients. In order to exclude the original CVD of patients with diabetes, we screened DFU patients with good venous valve function, normal venous reflux time, and no lower limb venous thrombosis to explore the effect of DFU on venous conditions. We measured their venous diameters in the lower limb and analyzed associated factors.

By understanding the venous conditions and their associated factors, we can enhance our predictive capabilities for identifying patients at risk, tailor therapeutic interventions more effectively, and ultimately improve patient outcomes. The significance of this research extends beyond clinical practice, as it contributes to the broader understanding of diabetes foot-related venous complications and their management.

## 2. Materials and Methods

### 2.1. Study Population and Design

A total number of 100 patients with DFU admitted in Sun Yat-sen Memorial Hospital from May 2021 to May 2024 were recruited. Exclusion criteria included (i) deep vein thrombosis of the lower limbs, (ii) varicose veins or reflux or venous valve disorder of the lower limbs, and (iii) concurrent pulmonary infection or other systemic infections. Clinical and biochemical characteristics were evaluated and compared between male and female groups, with most showing no significant differences, so did the diameters of lower extremity veins. Therefore, male and female data were merged for the following evaluation. The ratio of diameter of target vein between affected and unaffected side was calculated and represented the dilation level of the target vein. The protocol was approved by the institutional review board of Sun Yat-sen Memorial Hospital. Patient informed consent was waived due to the retrospective nature of the study. Confidentiality of patient information was maintained in accordance with the guidelines of the institutional review board.

### 2.2. Data Collection

The demographic features were collected via the clinical medical record system, including age, gender (male/female), duration of diabetes, side of foot ulcer lesion (left/right), and Wagner's grading of foot ulcer lesion. Body mass index (BMI), systolic blood pressure (SBP), and diastolic blood pressure (DBP) were collected during admission. Blood measurement for glucose-related parameters, inflammatory markers, and lipid parameters was performed within 2 days before or after ultrasound examination for the lower extremity veins. Glucose-related parameters included fasting blood glucose (FBG), hemoglobin A1c (HbA1c), fasting C-peptide, and ketone bodies, while inflammatory markers included high-sensitive C-reactive protein (hs-CRP), C-reactive protein (CRP), procalcitonin (PCT), positive bacterial culture of DFU secretions, white blood cell (WBC), platelet (PLT), and D-dimer. Total cholesterol (TC), triglycerides (TGs), nonesterified fatty acids (NEFAs), low-density lipoprotein (LDL), and high-density lipoprotein (HDL) were measured as lipid parameters. All the measurements were performed in the lab of Sun Yat-sen Memorial Hospital, and the elevated levels of ketones (≥ 3 mmol/L), hs-CRP (≥ 3 mg/L), CRP (≥ 10 mg/L), and PCT (≥ 0.1 ng/mL) were based on our lab standard.

### 2.3. Examination of Lower Extremity Veins and Dorsal Pedis Artery

Ultrasound examination of the lower extremity veins [8] and dorsal pedis artery for patients with DFU was performed by the same senior ultrasonographer with more than 15 years' experience. The GE logiq E20 ultrasound diagnostic instrument was used with a linear array probe, frequency 5–12 MHz. In order to exclude the original CVD of patients with diabetes, we screened DFU patients with good venous valve function, normal venous reflux time, and no lower limb venous thrombosis. All the patients enrolled had one side of DFU, which was defined as the affected side, while the other side was the unaffected side. Both sides of the common femoral vein (CFV), femoral vein (FV), great saphenous vein (GSV), and small saphenous vein (SSV) were examined, and their diameters were measured. The patients were in a supine position. The inner diameter of the CFV was measured at the proximal end of the saphenofemoral vein valve, while the inner diameter of the FV was measured above the first valve after bifurcation. Besides, the GSV was measured in transverse sections around the middle segment of the thigh and the middle segment of the calf. The diameter of the SSV was measured at the lower third of the back of the calf in a transverse section. Each pair of veins was measured at symmetrical positions on both sides. The anterior tibial vein, posterior tibial vein, and fibular vein often have two veins coexisting with the same named artery, and there is a lot of variation between the two veins, with differences in inner diameter, making it difficult to compare with the contralateral vein. Therefore, they were not measured in the current study.

The degree of ipsilateral dorsal pedis artery (iDPA) stenosis was graded using duplex ultrasound according to widely accepted criteria [9], which integrate spectral waveform analysis and peak systolic velocity ratios. Patients were classified into five categories based on the stenosis grade of the iDPA: none, mild, moderate, severe, and occlusion. However, in 20% of cases, assessment of the iDPA was not feasible due to dressing coverage over the diabetic foot wound (Table 1).

### 2.4. Statistical Analysis

Numerical variables and categorical variables were presented as mean ± standard deviation (SD) and number (percent) of patients, respectively. Independent samples *t*-test was performed for the comparison of numerical variables between male and female groups, while chi-square test or Fisher's exact test were used for the comparison of categorical variables between genders. In addition, paired *t*-test for numerical variables and McNemar's test for categorical variables were performed to compare the differences between two paired groups (affected side and unaffected side). The relationship between blood pressure, serum inflammatory markers, metabolic markers, and lower limb venous dilation was calculated by Spearman's rank correlation coefficient. Multiple linear regression analysis was subsequently used to confirm the independent factors related to lower limb venous dilation after adjusting for possible confounding variables. A two-sided *p* value of < 0.05 was considered statistically significant. All the data were analyzed using SPSS Version 26 (IBM, United States).

## 3. Results

### 3.1. Demographic and Clinical Characterization of the Cohort

A total of 100 target patients were included, with 72 males and 28 females. Their ages ranged from 30 to 90 years (mean 63.1 ± 12.8 years) with a duration of diabetes between 1 and 35 years (mean 12.1 ± 8.7 years). The patients had an average BMI of 23.43 ± 3.33 kg/m^2^ and an average HbA1c of 8.87% ± 2.15% (Table 1). The diabetic foot was rated using Wagner's grading system, Grades 2–4. There was no case of Grade 5 or Grades 0 and 1 recruited in the current study. Besides, patients were categorized according to the stenosis grade of the iDPA (none, mild, moderate, severe, and occlusion) (Table 1). In patients with DFU, the coincidence of positive bacterial culture was around 57%, with *Staphylococcus aureus* and *Proteus mirabilis* most commonly discovered (Table 1). A significant difference between male and female groups was found with regard to age (61 vs. 69; *p* = 0.02) and DBP (75 vs. 69; *p* = 0.02) (Table 1). However, the metabolic parameters, including both glucose and lipids as well as inflammatory markers measured, did not differ significantly between the two groups (Table 1). Also, there was no statistical difference with regard to diameters of lower extremity venous between male and female groups (Table 2). Therefore, both male and female data were merged for further correlation analysis.

### 3.2. Lower Extremity Venous Diameters of Patients With DFU

**Figure 1:**
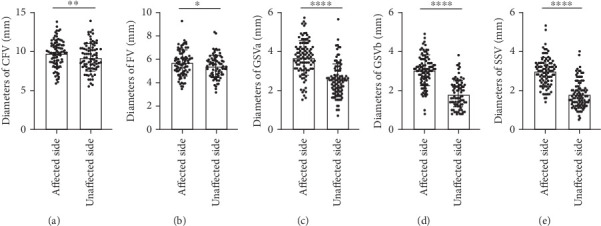
(a–e) Diameters of lower extremity venous of patients with diabetic foot ulcer. *p* value was calculated from paired *t*-test. ⁣^∗^*p* < 0.05, ⁣^∗∗^*p* < 0.01, and ⁣^∗∗∗∗^*p* < 0.0001. CFV, common femoral vein; FV, femoral vein; GSVa, great saphenous vein above knee; GSVb, great saphenous vein below knee; SSV, small saphenous vein.

All the patients enrolled were examined to exclude varicose veins and reflux of lower extremity venous or thrombosis. The incidence of bilateral lower limb foot ulcers in patients with diabetes is close to 1:1 (Table 1). Surprisingly, the diameters of both deep venous and superficial venous were wider in the ulcerated foot than those in the contralateral side (Table 2, *p* < 0.05) in the male group. In the female group, the diameters of superficial venous were wider in the ulcerated foot than those in the contralateral side, while the diameters of CFV and FV tended to be wider in the affected side (Table 2). In general, the venous dilation of CFV, FV, GSVa, GSVb, and SSV in the affected side compared to the unaffected side was observed in patients enrolled (Figure 1, *p* < 0.05). In addition, the tributaries and perforating veins of the GSV were more common in the affected side in the male group compared to the unaffected side, which did not reach statistical significance in the female group (Table 2). Furthermore, inguinal lymphadenopathy, usually a response to infection of an ipsilateral lower extremity [10], was indeed found in 73.6% of the male affected side and 60.7% of the female affected side (Table 2).

### 3.3. Correlation Analysis of the Ratio of Diameters of GSVa, GSVb, and SSV Between Affected and Unaffected Sides in Patients With DFU

The ratio of diameters of GSVa, GSVb, and SSV between affected and unaffected sides was calculated. Then, 90% of the ratio of GSVa was greater than 1, while 95% of the ratio of the GSVb and 98% of the ratio of SSV were greater than 1, suggesting superficial venous dilation in the ulcerated side of diabetic foot. In addition to male gender, duration of diabetes and FBG levels were positively correlated to the ratio of diameters of GSVb (*r* = 0.218, *p* = 0.033, Table 3). Furthermore, plasma levels of PCT (*r* = 0.231, *p* = 0.027) and WBC (*r* = 0.263, *p* = 0.011), markers of inflammation [11], were found to be positively correlated with the ratio of diameters of SSV (Table 3). Another possible factor is related to SBP (*r* = 0.331, *p* = 0.001). Interestingly, the plasma level of TG was negatively correlated to the ratio of diameters of both GSVa and GSVb (*r* = −0.222, *p* = 0.030 vs. *r* = −0.232, *p* = 0.027). Other metabolic parameters, including TC, NEFA, LDL, and HDL, did not contribute to the dilation of veins (Table 3). Furthermore, we observed no evidence of a correlation between the dilated veins and the Wagner grade of the foot ulcers or the plasma levels of PLT and D-dimer (Table 3). Similarly, the severity of arterial stenosis was not significantly associated with the diameter of the GSV or SSV in our analysis (Table 3). According to the above results of the Spearman correlation analysis, multiple linear regression analysis was performed to confirm the independent factor(s) that contributed to the venous dilation in patients with diabetes. Male gender and duration of diabetes were the positive predictors for the changes of the ratio of diameters of GSVb after correction for the age, DBP, and plasma levels of FBG and TG (Table 4). Besides, SBP was an independent positive predictor for the changes of the ratio of diameters of SSV after adjustment for the gender, plasma levels of PCT, and WBC (Table 4).

## 4. Discussion

Previous studies had indicated a higher occurrence of chronic venous insufficiency in the diabetic foot patients [6, 7], as evidenced by lower limb valvular incompetence, varicosity, and thrombosis, which might exacerbate leg ulcers. However, venous insufficiency occurs before or after the occurrence of DFU, which is difficult to distinguish in most patients with diabetes. In this study, we found out that venous dilation displayed in the affected side of CFV, FV, GSVa, and GSVb, as well as SSV after excluding CVD in patients with DFU. This might suggest the early change in venous structure in DFU. Furthermore, we sought to explore the independent predictor of venous dilation in the affected side of DFU.

Several risk factors have been identified for venous dysfunction in the general population, including age, female, obesity, and high blood pressure [5, 12]. Therefore, age, gender, BMI, and both SBP and DBP of the recruited patients were collected. To investigate the potential link between arterial insufficiency and venous remodeling, patients were categorized according to the stenosis grade of the iDPA. Besides, a proinflammatory condition is typical in diabetes [13], where hyperglycemia induces oxidative stress and increases the adhesion of leukocytes to the endothelium via the release of proteolytic enzymes, elevating local inflammation of the vessel walls, which may accelerate venous changes [14]. Serum inflammatory markers including WBC, hs-CRP, CRP, and PCT [15] were subsequently measured for further analysis. What is more, serum metabolic markers for glucose and lipids were detected [15], such as FBG, HbA1c, LDL, and HDL.

In the current study, the enrolled participants were aged around 60.7 years old in males and 69.3 years old in females. They have an average duration of diabetes of over a decade, over half of whom had Wagner Grade 4 foot ulcers. In addition, 45% of the enrolled patients had varying degrees of dorsal pedis artery stenosis. Regarding the parameters for glucose control, plasma lipids and cholesterol, as well as inflammation, the male and female groups did not show significant differences. Therefore, the ratio of affected side and unaffected side of lower extremity superficial venous diameter size was calculated in all enrolled patients.

It was not surprising to find that the duration of diabetes and FBG were positively correlated with the ratio of diameters of GSVb. With a longer history of diabetes or higher FBG, it is more likely to have dilated GSV in the affected calf. Besides, plasma TG appeared to be negatively correlated with the ratio of both GSVa and GSVb. One reason could be that all the enrolled patients had a normal range of plasma TG, and lower plasma TG levels among them might suggest poorer nutritional status due to the dysglycemia [16]. Therefore, with a lower plasma TG level, it is more likely to have dilated GSV in the affected leg. As for the ratio of SSV, SBP, plasma levels of PCT, and WBC were found to show a positive correlation. It indicated that systemic inflammation might contribute to the expanded SSV in the affected leg. Similar to previous studies, DFUs were mainly colonized by *Staphylococcus aureus* and *Proteus mirabilis* [14, 17]. However, the wound site microbiome did not seem to show significant correlations to dilated veins. The interplay between blood pressure, serum inflammatory markers, and metabolic markers in these patients is complex and might influence the progression of venous disease. Furthermore, our study did not identify a significant correlation between Wagner's severity and the extent of venous dilation. This may suggest that venous dilation represents an early, independent component of diabetic vasculopathy, establishing a pathological environment conducive to ulcer formation without its severity being directly proportional to the ultimate depth or infectious complication of the ulcer, as captured by the Wagner classification.

After adjusting for the possible associations, SBP was an independent predictor for the ratio of SSV, and higher levels of SBP tended to be the independent predictor for the ratio of GSVa. Elevated blood pressure may exacerbate the hemodynamic stress on the venous system. It demonstrated that with a better blood pressure control, it may reduce the risk of venous dysfunction in patients with DFU. Our study did not demonstrate a significant correlation between the severity of iDPA stenosis and the degree of GSV and SSV dilation in patients with DFUs. The absence of a link between local arterial stenosis and venous dilation suggests that the venous remodeling process in DFU may not be primarily driven by localized hemodynamic changes downstream of a specific arterial occlusion. Instead, the significant positive correlation with systemic blood pressure points toward a systemic, rather than a local, pathological mechanism. This implies that factors affecting the entire cardiovascular system are more influential in causing venous dilation than the status of a single peripheral artery. In addition, male sex and duration of diabetes were independent predictors for the ratio of GSVb. Although one would expect elevated inflammation might predict the dilated veins [18], plasma PCT and WBC levels did not show significant results. This is likely due to the fact that metabolic inflammation from diabetes and venous inflammation from the DFU were represented by the plasma inflammatory parameters, which did not point to the venous dysfunction only.

There are several limitations that need to be highlighted in the current study. Firstly, it is a retrospective study with a relatively small sample size. Secondly, the enrolled diabetic foot patients were graded between 2 and 4. Future research with a larger and more diverse samples should be conducted. Thirdly, we cannot establish causality from our observations with correlation and regression analysis. Follow-up studies concerning the changes in venous structure and function after dilation are required and might clarify the progress of venous disease in DFU. Finally, it is possible that our method of grading DPA stenosis may not fully capture the functional hemodynamic significance of the stenosis. Techniques such as measurement of distal perfusion pressure or transcutaneous oxygen pressure (TcPO_2_) might provide a more physiologically accurate assessment of the ischemic burden that could influence venous remodeling.

We may summarize that venous dilation is very common in the lower extremity superficial veins on the affected side of DFU after excluding concurrent CVD. Although plasma PCT and WBC levels showed a positive association with the ratio of SSV, they were not independent factors. Venous dilation in DFU is a manifestation of systemic cardiovascular pathology linked to hypertension, rather than a direct compensatory response to localized arterial stenosis. SBP showed independent prediction for the ratio of GSV and SSV, suggesting the importance of SBP control in patients with DFU. In light of the escalating healthcare burden in DFU, the exploration of the venous condition in DFU is not just a clinical necessity but a critical step toward comprehensive diabetes care.

## Figures and Tables

**Table 1:** Baseline characteristics of patients with diabetic foot ulcer.

**Variables**	**Male (** **n** = 72**)**	**Female (** **n** = 28**)**	**p** **value**
Age (years)	60.7 ± 12.8	69.3 ± 10.5	**0.02**
BMI (kg/m^2^)	23.79 ± 3.36	22.38 ± 3.08	> 0.05
SBP (mmHg)	136 ± 22	138 ± 22	> 0.05
DBP (mmHg)	75 ± 13	69 ± 10	**0.02**
Diabetes duration (years)	11.7 ± 8.3	13.3 ± 9.5	> 0.05
< 5 years	17 (23.6%)	7 (25%)	
5–10 years	19 (26.4%)	5 (17.9%)	
> 10 years	36 (50%)	16 (57.1%)	
Ulcer lesion			> 0.05
Left	32 (44.4%)	16 (57.1%)	
Right	40 (55.6%)	12 (42.9%)	
Wagner's grading			> 0.05
2–3	28 (38.9%)	8 (28.6%)	
4	44 (61.1%)	20 (71.4%)	
Degree of iDPA stenosis			> 0.05
NA^a^	13 (18.1%)	7 (25%)	
None	28 (38.9%)	7 (25%)	
Mild	11 (15.3%)	5 (17.9%)	
Moderate	8 (11.1%)	4 (14.3%)	
Severe	11 (15.3%)	2 (7.1%)	
Occlusion	1 (1.4%)	3 (10.7%)	
FBG (mmol/L)	8.81 ± 4.11	10.41 ± 4.40	> 0.05
HbA1c (%)	8.78 ± 2.11	9.09 ± 2.29	> 0.05
Fasting C-peptide (pmol/L)	554.10 ± 452.65	613.23 ± 539.25	> 0.05
Ketones ≥ 3 mmol/L	11 (15.3%)	6 (21.4%)	> 0.05
TC (mmol/L)	3.77 ± 1.01	4.05 ± 1.19	> 0.05
TG (mmol/L)	1.17 ± 0.52	1.33 ± 0.64	> 0.05
NEFA (mmol/L)	369.29 ± 181.15	383.60 ± 182.00	> 0.05
LDL (mmol/L)	2.31 ± 0.76	2.46 ± 0.75	> 0.05
HDL (mmol/L)	0.84 ± 0.23	0.91 ± 0.35	> 0.05
hs‐CRP ≥ 3 mg/L	67 (93.1%)	24 (85.7%)	> 0.05
CRP ≥ 10 mg/L	57 (79.2%)	21 (75%)	> 0.05
PCT ≥ 0.1 ng/mL	21 (29.2%)	9 (32.1%)	> 0.05
Positive bacterial culture	46 (63.9%)	11 (39.3%)	> 0.05
WBC (10^9^/L)	11.66 ± 7.95	12.67 ± 4.95	> 0.05
PLT (10^9^/L)	328.13 ± 96.79	371.04 ± 110.67	> 0.05
D-dimer (mg/L)	1.24 ± 1.12	2.00 ± 4.00	> 0.05

*Note:* Data are shown as mean ± SD or *n* (percent). *p* value refers to the comparison of male and female groups by independent samples *t*-test, chi-square test, or Fisher's exact test. A *p* value of < 0.05 was considered statistical significance, which was shown in bold.

Abbreviations: BMI, body mass index; CRP, C-reactive protein; DBP, diastolic blood pressure; FBG, fasting blood glucose; HbA1c, hemoglobin A1c; HDL, high-density lipoprotein; hs-CRP, high-sensitive C-reactive protein; iDPA, ipsilateral dorsal pedis artery; LDL, low-density lipoprotein; NEFAs, nonesterified fatty acids; PCT, procalcitonin; PLT, platelet; SBP, systolic blood pressure; TC, total cholesterol; TGs, triglycerides; WBC, white blood cell.

^a^NA: the condition of the dorsal pedis artery could not be assessed due to the dressing coverage on the patient's diabetic foot wound.

**Table 2:** Lower extremity venous conditions of patients with diabetic foot ulcers.

**Variables**	**Male (** **n** = 72**)**	**Female (** **n** = 28**)**
Common femoral vein diameter (mm)		
Affected side	9.9 ± 1.8	9.5 ± 1.7
Unaffected side	9.2 ± 1.8	9.2 ± 1.6
*p* value	**0.003**	0.266
Femoral vein diameter (mm)		
Affected side	5.8 ± 1.1	5.5 ± 0.9
Unaffected side	5.4 ± 1.0	5.3 ± 0.9
*p* value	**0.027**	0.238
Diameter of great saphenous vein above knee (mm)		
Affected side	3.7 ± 0.8	3.5 ± 1.0
Unaffected side	2.6 ± 0.9	2.3 ± 0.7
*p* value	**< 0.001**	**< 0.001**
Diameter of great saphenous vein below knee (mm)		
Affected side	3.1 ± 0.8	2.9 ± 0.7
Unaffected side	2.0 ± 0.6	1.5 ± 0.6
*p* value	**< 0.001**	**< 0.001**
With tributaries of the great saphenous vein		
Affected side	28 (38.9)	14 (50.0)
Unaffected side	9 (12.5)	8 (28.6)
*p* value	**< 0.001**	0.125
With perforating veins of the great saphenous vein		
Affected side	13 (18.1)	8 (28.6)
Unaffected side	6 (8.3)	2 (7.1)
*p* value	**0.016**	0.063
Small saphenous vein diameter (mm)		
Affected side	3.0 ± 0.7	2.7 ± 0.7
Unaffected side	1.9 ± 0.7	1.5 ± 0.5
*p* value	**< 0.001**	**< 0.001**
With tributaries of the small saphenous vein		
Affected side	11 (15.3)	5 (17.9)
Unaffected side	8 (11.1)	2 (7.1)
*p* value	0.581	0.250
With perforating veins of the small saphenous vein		
Affected side	4 (5.6)	1 (3.6)
Unaffected side	1 (1.4)	1 (3.6)
*p* value	/	/
Inguinal lymphadenopathy		
Affected side	53 (73.6)	17 (60.7)
Unaffected side	7 (9.7)	3 (10.7)
*p* value	**< 0.001**	**< 0.001**

*Note: p* value refers to the comparison of affected and unaffected sides by pair *t*-test or McNemar's test within the same gender. A *p* value of < 0.05 was considered statistical significance, which was shown in bold.

**Table 3:** Correlation analysis of the ratio of lower extremity superficial venous diameter size in patients with diabetic foot ulcer.

	**The ratio of diameters of GSVa**	**The ratio of diameters of GSVb**	**The ratio of diameters of SSV**
Age (years)			
*r*	−0.001	0.191	0.077
*p*	0.995	0.063	0.461
Gender (M/F)			
*r*	0.011	**0.222**	0.177
*p*	0.915	**0.030**	0.089
Diabetes duration (years)			
*r*	0.034	**0.265**	0.030
*p*	0.738	**0.009**	0.778
Diabetes duration group (< 5 years, 5–10 years, and > 10 years)			
*r*	0.018	0.168	0.085
*p*	0.862	0.101	0.419
Wagner's grading			
*r*	0.194	−0.011	0.038
*p*	0.053	0.913	0.715
BMI (kg/m^2^)			
*r*	0.011	−0.067	−0.130
*p*	0.922	0.541	0.241
SBP (mmHg)			
*r*	0.193	0.077	**0.331**
*p*	0.054	0.458	**0.001**
DBP (mmHg)			
*r*	0.068	−0.194	0.054
*p*	0.500	0.059	0.604
Degree of iDPA stenosis			
*r*	0.016	0.057	0.008
*p*	0.890	0.621	0.943
FBG (mmol/L)			
*r*	0.002	**0.218**	0.050
*p*	0.984	**0.033**	0.637
Fasting C-peptide (pmol/L)			
*r*	−0.003	−0.002	−0.009
*p*	0.979	0.988	0.938
Ketone bodies (mmol/L)			
*r*	−0.014	−0.116	0.003
*p*	0.895	0.278	0.981
HbA1c (%)			
*r*	−0.170	0.036	0.121
*p*	0.092	0.730	0.250
Procalcitonin (ng/mL)			
*r*	0.082	0.113	**0.231**
*p*	0.422	0.280	**0.027**
CRP ≥ 10 mg/L			
*r*	0.130	−0.106	0.047
*p*	0.208	0.312	0.659
hs‐CRP ≥ 3 mg/L			
*r*	0.028	−0.107	−0.148
*p*	0.789	0.311	0.163
WBC (10^9^/L)			
*r*	0.136	0.110	**0.263**
*p*	0.178	0.285	**0.011**
Positive bacterial culture			
*r*	0.078	0.016	−0.003
*p*	0.442	0.875	0.975
PLT (10^9^/L)			
*r*	0.095	−0.042	0.131
*p*	0.345	0.682	0.212
D-dimer (mg/L)			
*r*	−0.120	−0.023	0.145
*p*	0.234	0.827	0.167
TG (mmol/L)			
*r*	**−0.222**	**−0.232**	−0.047
*p*	**0.030**	**0.027**	0.666
TC (mmol/L)			
*r*	−0.014	−0.053	−0.158
*p*	0.895	0.615	0.141
NEFA (mmol/L)			
*r*	−0.184	0.069	0.137
*p*	0.093	0.546	0.235
LDL (mmol/L)			
*r*	−0.019	−0.069	−0.104
*p*	0.853	0.517	0.336
HDL (mmol/L)			
*r*	−0.066	0.056	−0.150
*p*	0.525	0.598	0.164

*Note:* Correlation analysis of the ratio of diameters of GSVa, GSVb, and SSV between affected and unaffected sides in patients with diabetic foot ulcer was performed. Shown are *r* and *p* values from Spearman's analysis. A *p* value of < 0.05 was considered statistical significance, which was shown in bold.

Abbreviations: BMI, body mass index; CRP, C-reactive protein; DBP, diastolic blood pressure; F, female; FBG, fasting blood glucose; GSVa, great saphenous vein above knee; GSVb, great saphenous vein below knee; HbA1c, hemoglobin A1c; HDL, high-density lipoprotein; hs-CRP, high-sensitive C-reactive protein; iDPA, ipsilateral dorsal pedis artery; LDL, low-density lipoprotein; M, male; NEFAs, nonesterified fatty acids; PLT, platelet; SBP, systolic blood pressure; SSV, small saphenous vein; TC, total cholesterol; TGs, triglycerides; WBC, white blood cell.

**Table 4:** Multiple linear regression analysis of the ratio of lower extremity superficial venous diameter size in patients with diabetic foot ulcer.

	**Standardized coefficient *β***	**t**	**p** **value**
The ratio of diameters of GSVa between affected and unaffected side			
SBP > 140 mmHg	0.198	1.947	0.055
TG (mmol/L)	−0.171	−1.671	0.098
Wagner's grading	0.119	1.151	0.253
HbA1c (%)	−0.130	−1.262	0.210
The ratio of diameters of GSVb between affected and unaffected side			
Gender (male/female)	**0.264**	**2.643**	**0.010**
Duration of diabetes (years)	**0.224**	**2.242**	**0.027**
Age (years)	0.060	0.542	0.589
Diastolic blood pressure (mmHg)	−0.077	−0.745	0.458
Fasting blood glucose (mmol/L)	0.078	0.741	0.461
TG (mmol/L)	−0.039	−0.385	0.701
The ratio of diameters of SSV between affected and unaffected side			
Systolic blood pressure (mmHg)	**0.229**	**2.221**	**0.029**
Gender (male/female)	0.141	1.394	0.167
Procalcitonin (ng/mL)	0.137	1.294	0.199
WBC (10^9^/L)	0.130	1.196	0.235

*Note: p* value were calculated using multiple linear regression analysis, with statistically significant results highlighted in bold.

Abbreviations: HbA1c, hemoglobin A1c; SBP, systolic blood pressure; TGs, triglycerides; WBC, white blood cell.

## Data Availability

The original datasets analyzed in the current study are available from the corresponding author upon reasonable request.

## References

[1] Ouyang W., Jia Y., Jin L. (2021). Risk Factors of Diabetic Foot Ulcer in Patients With Type 2 Diabetes: A Retrospective Cohort Study. *American Journal of Translational Research*.

[2] Armstrong D. G., Tan T. W., Boulton A., Bus S. A. (2023). Diabetic Foot Ulcers. *Journal of the American Medical Association*.

[3] Deng H., Li B., Shen Q. (2023). Mechanisms of Diabetic Foot Ulceration: A Review. *Journal of Diabetes*.

[4] Krizanova O., Penesova A., Hokynkova A., Pokorna A., Samadian A., Babula P. (2024). Chronic Venous Insufficiency and Venous Leg Ulcers: Aetiology, on the Pathophysiology-Based Treatment. *International Wound Journal*.

[5] Jarosikova R., Roztocil K., Husakova J. (2023). Chronic Venous Disease and Its Intersections With Diabetes Mellitus. *Physiological Research*.

[6] Gastaldi G., Pannier F., Roztocil K. (2021). Chronic Venous Disease and Diabetic Microangiopathy: Pathophysiology and Commonalities. *International Angiology*.

[7] Fejfarova V., Roztocil K., Svedinkova A. (2017). The Relationship Between Chronic Venous Insufficiency and Diabetes Mellitus. *International Angiology*.

[8] Shabani V. E., Gargiulo G. D., Penkala S., Breen P. P. (2018). Peripheral Vascular Disease Assessment in the Lower Limb: A Review of Current and Emerging Non-Invasive Diagnostic Methods. *Biomedical Engineering Online*.

[9] Koelemay M. J., den Hartog D., Prins M. H., Kromhout J. G., Legemate D. A., Jacobs M. J. (1996). Diagnosis of Arterial Disease of the Lower Extremities With Duplex Ultrasonography. *British Journal of Surgery*.

[10] Qin L., Zhao C., Wang H. (2023). Detection of Inguinal Lymph Nodes Is Promising for the Diagnosis of Periprosthetic Joint Infection. *Frontiers in Cellular and Infection Microbiology*.

[11] Tan M., Lu Y., Jiang H., Zhang L. (2019). The Diagnostic Accuracy of Procalcitonin and C-Reactive Protein for Sepsis: A Systematic Review and Meta-Analysis. *Journal of Cellular Biochemistry*.

[12] Criqui M. H., Denenberg J. O., Bergan J., Langer R. D., Fronek A. (2007). Risk Factors for Chronic Venous Disease: The San Diego Population Study. *Journal of Vascular Surgery*.

[13] Li R., Lu B., Li Q. (2024). Characteristics of Metabolic Inflammatory Syndrome Among Inpatients With Type 2 Diabetes: A Cross-Sectional Study in China. *Primary Care Diabetes*.

[14] Castro-Ferreira R., Cardoso R., Leite-Moreira A., Mansilha A. (2018). The Role of Endothelial Dysfunction and Inflammation in Chronic Venous Disease. *Annals of Vascular Surgery*.

[15] Wang Y., Shao T., Wang J. (2021). An Update on Potential Biomarkers for Diagnosing Diabetic Foot Ulcer at Early Stage. *Biomedicine & Pharmacotherapy*.

[16] Feingold K. R. (2023). The Bidirectional Interaction of COVID-19 Infections and Lipoproteins. *Best Practice & Research. Clinical Endocrinology & Metabolism*.

[17] Walczak-Skierska J., Monedeiro F., Maslak E., Zloch M. (2023). Lipidomics Characterization of the Microbiome in People With Diabetic Foot Infection Using MALDI-TOF MS. *Analytical Chemistry*.

[18] MacColl E., Khalil R. A. (2015). Matrix Metalloproteinases as Regulators of Vein Structure and Function: Implications in Chronic Venous Disease. *Journal of Pharmacology and Experimental Therapeutics*.

